# Occupational Electromagnetic Field Exposures Associated with Sleep Quality: A Cross-Sectional Study

**DOI:** 10.1371/journal.pone.0110825

**Published:** 2014-10-23

**Authors:** Hui Liu, Guangdi Chen, Yifeng Pan, Zexin Chen, Wen Jin, Chuan Sun, Chunjing Chen, Xuanjun Dong, Kun Chen, Zhengping Xu, Shanchun Zhang, Yunxian Yu

**Affiliations:** 1 Department of Epidemiology & Health Statistics, School of Public Health, School of Medicine, Zhejiang University, Hangzhou, Zhejiang, China; 2 Bioelectromagnetics Laboratory, School of Medicine, Zhejiang University, Hangzhou, Zhejiang, China; 3 Chronic Disease Research Institute, School of Public Health, School of Medicine, Zhejiang University, Hangzhou, Zhejiang, China; 4 Yiwu Center for Disease Control and Prevention, Yiwu, Zhejiang, China; “Mario Negri” Institute for Pharmacological Research, Italy

## Abstract

**Background:**

Exposure to electromagnetic field (EMF) emitted by mobile phone and other machineries concerns half the world’s population and raises the problem of their impact on human health. The present study aims to explore the effects of electromagnetic field exposures on sleep quality and sleep duration among workers from electric power plant.

**Methods:**

A cross-sectional study was conducted in an electric power plant of Zhejiang Province, China. A total of 854 participants were included in the final analysis. The detailed information of participants was obtained by trained investigators using a structured questionnaire, which including socio-demographic characteristics, lifestyle variables, sleep variables and electromagnetic exposures. Physical examination and venous blood collection were also carried out for every study subject.

**Results:**

After grouping daily occupational electromagnetic exposure into three categories, subjects with long daily exposure time had a significantly higher risk of poor sleep quality in comparison to those with short daily exposure time. The adjusted odds ratios were 1.68 (95%CI: 1.18, 2.39) and 1.57 (95%CI: 1.10, 2.24) across tertiles. Additionally, among the subjects with long-term occupational exposure, the longer daily occupational exposure time apparently increased the risk of poor sleep quality (OR (95%CI): 2.12 (1.23∼3.66) in the second tertile; 1.83 (1.07∼3.15) in the third tertile). There was no significant association of long-term occupational exposure duration, monthly electric fee or years of mobile-phone use with sleep quality or sleep duration.

**Conclusions:**

The findings showed that daily occupational EMF exposure was positively associated with poor sleep quality. It implies EMF exposure may damage human sleep quality rather than sleep duration.

## Introduction

Previous epidemiologic studies have suggested that sleep disorder plays an important role in overall human health and diseases including depression and anxiety [1], obesity [2], metabolic syndrome [3]–[5], diabetes [6], and immune function [7]. Furthermore, poor sleep quality even augments the risk of all-cause mortality [8]. Besides, the prevalence of sleep disturbances is pervasive, 25.6% in adolescents [9], and 57.1% in a large middle-aged Colombian female population [10]. In China, the prevalence of sleep disorder was 21.9% for urban children aged 0 to 23 months [11]. For Chinese older community-dwelling individuals, most participants (nearly 77%) were poor sleepers [12].

In the recent decades, emerging wireless technologies like mobile phones or cordless phones have become ubiquitous. It is an important source for individuals’ radiofrequency electromagnetic fields exposures (RF-EMF) in daily life [13]. According to a report of the World Health Organization (WHO), the intensity of spatial EMF has expanded enormously due to the increase of cellular wireless technology [14]. Several publications have raised concerns about the individual and public health impact of adverse non-ionizing radiation from electromagnetic field (EMF) exposure emanating from certain power, electrical and wireless devices commonly found in the home, workplace, school and community [15]. Although previous studies have indicated that any potential cellular and tissue damage is associated with exposure to ionizing radiation from X-rays, electromagnetic field (EMF) emitting from power lines, recent attention has suggested that mobile phones, interphones, common electrical devices and some types of machinery are potential health hazards. But, causal relationships between wireless phone use and the incidence of human diseases, such as brain tumors, cardiovascular disease, and adverse reproductive outcomes, have not been established consistently [16]–[19].

Although most research focused on the association between brain tumorigenesis and low-intensity electromagnetic radiation, studies regarding the relationship of sleep disturbances and RF-EMF exposure have also emerged recently. Schreier et al [20] reported that sleep disturbances were the most common health complaints attributed to RF-EMF exposure in the general population of Switzerland. Furthermore, some laboratory studies demonstrated that electromagnetic field exposure prior to sleep increased the power in the spindle frequency range during sleep stage 2 of non-REM sleep in the first few hours of sleep [21], [22]. Nonetheless, the apparent associations of EMF exposure with adverse sleep quality were not observed in several population studies [23]–[26].

Additionally, epidemiological studies regarding electromagnetic exposure effects on sleep quality or sleep duration were conducted mainly in western populations. Yet to date, few similar studies were performed in Chinese populations. The Chinese population comprises a fifth of the world’s population and is undergoing rapid economic growth and lifestyle changes, which are accompanied by rapidly rising rates of sleep disturbance and related chronic diseases, such as obesity, type 2 diabetes, and MS. Therefore, the causes of sleep disturbance have to be explored for improving sleep quality. The purpose of this study was to discover a probable relationship between self-assessed sleep status and electromagnetic field exposures, especially power frequency magnetic fields, in workers of a power plant.

## Materials and Methods

### Study design and participants

Participants were enrolled in an electric power plant of Zhejiang province, China since August to September 2011. In total, 1073 individuals, containing 863 males and 210 females aged between 22 to 60 years were recruited. We excluded individuals who had retired, or had any type of cancer, severe heart, liver or kidney disease. Since some participants did not provide information about occupational electromagnetic field exposure, 854 participants were included into the final analysis. The study protocol was approved by the Medical Ethical Committee of Zhejiang University School of Medicine.

After written informed consent was obtained from each subject, a face-to-face interview was conducted by well-trained medical students using a structured questionnaire including socio-demographic characteristics (e.g. age, gender, department, marital status and education level), lifestyle (e.g. cigarette smoking, alcohol drinking and tea drinking) and electromagnetic radiation exposures (e.g. occupational exposure hours per day, years of mobile-phone service, electric fee in family per month and occupational exposure duration).

Health check-ups including internal medicine examination, surgery examination, blood routine, urine routine and hepatic function routine were conducted for each participant. Furthermore, 3 ml peripheral vein blood was collected using a vacuum tube containing Ethylene Diamine Tetraacetic Acid (***EDTA***) anticoagulation from each subject and stored in a −80°C refrigerator.

### Sleep parameter assessments

Sleep quality was assessed using the question “How do you assess the quality of your sleep during the past one month?” Three options were provided: Good, Fair and Bad. In Chinese culture, the answer of “Fair” for any question means “not so good”. Therefore, if the answer was “Fair” or “Bad”, the individual would be defined as having “subjective poor sleep quality”. Otherwise, subjects who answered “Good” were considered as having “subjective good sleep quality”. The question used for sleep duration assessment was “How many hours on average have you slept at night during the past one month?” If the sleep duration was <7 h, 7 h∼ and ≥8 h then the sleep duration was grouped as “short sleep duration”, “medium sleep duration” and “long sleep duration”, respectively.

### Electromagnetic field exposure assessments

The electromagnetic field exposure level of participants was measured by the electromagnetic field exposure record of the employer and a series of relevant questions. Occupational EMF exposure was assessed on the basis of job titles, job description, the conventional measured data of occupational EMF from the electric power plant and walkie talkies usage each day. In order to confirm whether it was appropriate for the assessment of occupational EMF exposure provided by the power plant, we measured intensity of occupational EMF in some main activity areas of workers, such as office, rest room and workshop. Intensity of occupational EMF was measured at each measuring site according to environmental protection industry standards of the People’s Republic of China (1996), which named the Guidelines on Management of Radioactive Environmental Protection Electromagnetic Radiation Monitoring Instruments and Methods (HJ/T10.2–96). Intensity of occupational EMF was measured with an EFA-300 meter (Narda Safety Test Solutions GmbH, Pfullingen, Germany) for the low frequency range (5 Hz–32 kHz). EFA-300 is equipped with three-dimensional isotropic measurement probe (non-directional). The electric and magnetic field ranges of the instrument are 0.1 V/m–316 kV/m and 0.1 nT–32 mT, respectively, with a typical accuracy at ±3%. The measuring probe was consistently placed at a height of 1.5 m from the ground. Both electric field intensity in V/m and magnetic field intensity in nT of 50 Hz were measured. Before the electric and magnetic field measurements were carried out, the EFA-300 meter was calibrated, and linearity and frequency responses were checked. Each spot measurement was acquired over a minimum period of 15 s. Upon stabilization of a reading, the maximum value was recorded. Continuous measurements were performed in quintuplicate or sextuplicate at each measuring site, and the average was expressed as the means ± SD. The intensity of occupational EMF in the exposure group was (316.3±1212.3) V/m for electric fields and (6171.9±14713.1) nT for magnetic fields, which far outstripped that in non-exposure group ((3.9±0.3) V/m and (60.6±16.9) nT, respectively).

The EMF exposures of each participant were assessed by a series of relevant questions. Daily occupational exposure time was assessed using the question, “How many hours per day on average are you exposed to electrical equipment in the workplace?” We classified daily occupational exposure time into tertiles. The question about occupational exposure duration was, “How many years have you engaged in this job in the power plant?” In the analysis, the variable of working duration was transformed into a binary variable according to the median. Question – “How much is your monthly electrical bill on average?” was used to approximately assess electromagnetic radiation exposure at home. We also used the median to classify the variable into a binary variable. With regard to cell phone usage, “How many years have you been using a mobile phone” was used to evaluate the EMF exposure from cell phone use. Similarly, the data were split at the median into a binary variable.

### Work stress assessments

The question “How was your work stress during the past one month?” was used to assess the work stress of participants. The four options were provided: None, Low, Medium and High. Due to the small sample size in the none-work stress and high work stress groups, we combined none and low work stress groups as the low work stress or none group. Accordingly, the medium and high work stress groups were merged into the medium work stress or more group.

### Statistical analysis

The distribution of socio-demographic characteristics between different exposure groups was tested using Student’s t-test for the continuous variables and Pearson’s χ^2^ test for the categorical variables. The associations of various electromagnetic exposures (such as daily occupational electromagnetic radiation exposure time, occupational electromagnetic exposure duration, etc.) with sleep quality and sleep duration were conducted, respectively. A logistic regression model was used to assess these associations with and without adjustment for potential confounding factors, including age (continuous), sex (binary), smoke (binary), tea drinking (binary), BMI (continuous), and work stress (binary). Additionally, stratified analysis was used to assess the association of occupational exposure duration and daily occupational exposure time on the sleep quality and duration. Odds ratios were calculated from both univariate logistic regression analysis and multivariate logistic regression analysis with 95% CIs. The statistical significance threshold was a two-sided *P* value equal to or less than 0.05. All analyses were performed using Statistical Analysis System software version 9.2 (SAS Institute Inc, Cary, North Carolina).

## Results

### Description of general population

Table 1 shows the distributions of socio-demographic characteristics between good sleepers and poor sleepers. 323 individuals with good sleep quality and 531 with poor sleep quality were recruited in the final analysis. Good sleepers had a higher BMI in comparison with poor sleepers. The distributions of gender, marital status, education, total income per family, alcohol drinking, hypertension and diabetes were comparable between the two groups. However, the distributions of cigarette smoking, tea drinking, and work stress between poor and good sleepers were statistically significant difference.

**Table 1:** Demographic characteristics of study subjects stratified by sleep quality.

Characteristics	Sleep quality, N (%)		*P* value^b^
	Poor (N = 531)	Good (N = 323)	
**Age, years (Mean ± SD)**	43.1±7.4	43.6±7.4	0.366
**BMI, Kg/m^2^ (Mean ± SD)**	23.0±3.0	23.7±2.8	**0.001**
**Gender**			0.873
Male	435 (81.9)	266 (82.4)	
Female	96 (18.1)	57 (17.6)	
**Marital Status**			0.922
Married	499 (94.0)	303 (93.8)	
Unmarried^a^	32 (6.0)	20 (6.2)	
**Education**			0.324
Senior High School and Below	252 (47.5)	140 (43.3)	
College	193 (36.3)	119 (36.8)	
University and Above	86 (16.2)	64 (19.8)	
**Income (10,000 yuan/year)**			0.083
<1	67 (12.6)	25 (7.7)	
1–3	78 (14.7)	49 (15.2)	
≥3	386 (72.7)	249 (77.1)	
**Cigarette Smoking**			**0.026**
No	298 (56.1)	156 (48.3)	
Yes	233 (43.9)	167 (51.7)	
**Alcohol Drinking**			0.454
No	397 (74.8)	234 (72.4)	
Yes	134 (25.2)	89 (27.6)	
**Tea Drinking**			**0.003**
No	284 (53.5)	139 (43.0)	
Yes	247 (46.5)	184 (57.0)	
**Work Stress**			**0.001**
Low or none	285 (53.7)	210 (65.0)	
Medium or more	246 (46.3)	113 (35.0)	

a The unmarried include those who are single, divorced or widowed.

b Comparison between groups was tested used Student’s *t*-test for the continuous variables and Pearson’s χ^2^ test for the categorical variables.

### Association of EMF exposure with sleep quality and sleep duration

The associations of EMF exposures with sleep quality were presented in Table 2. Across the trisections, workers with longer daily occupational electromagnetic exposure time had a significantly higher risk of poor sleep quality (T2: Adjusted OR = 1.68, 95%CI: 1.18, 2.39; T3: Adjusted OR = 1.57, 95%CI: 1.10, 2.24) compared with those with short daily EMF exposure time. No notable associations of occupational exposure duration, monthly electric fee or years of mobile-phone service with sleep quality were detected. Based on the EMF record of the employer, exposure of workers to electromagnetic fields in workplaces did not increase the risk of poor sleep, compared with those who were not exposed to electromagnetic fields (OR = 1.33, 95%CI: 0.94, 1.89) (data not shown). However, there was a strong relationship between electromagnetic field exposure and sleep quality among participants whose self-reported EMF exposure information was consistent with the EMF exposure record of the employer with adjustment (OR = 1.81, 95%CI: 1.04, 3.16) (data not shown). After adjusting for diseases that may affect sleep quality such as hypertension and diabetes, the finding regarding risk to sleep quality from electromagnetic fields was similar (data not shown).

**Table 2:** Associations of sleep quality with various electromagnetic exposures.

Variables		Sleep Quality		Crude		Adjusted^a^	
		Poor, N (%)	Good, N (%)	OR (95%CI)	*P* Value	OR (95%CI)	*P* Value
**Daily Occupational Exposure Time** (hours)^b^	T1	150 (28.2)	132 (40.9)	1.00	*---*	1.00	*---*
	T2	194 (36.5)	94 (29.1)	1.82 (1.29, 2.55)	**<0.001**	1.68 (1.18, 2.39)	**0.004**
	T3	187 (35.2)	97 (30.0)	1.70 (1.21, 2.38)	**0.002**	1.57 (1.10, 2.24)	**0.012**
**Occupational Exposure Duration** (years)	<23	270 (50.8)	177 (54.8)	1.00	*---*	1.00	*---*
	≥23	261 (49.2)	146 (45.2)	1.17 (0.89, 1.55)	0.262	1.26 (0.91, 1.76)	0.167
**Electric fee** (yuan/month)	<150	207 (39.0)	129 (39.9)	1.00	*---*	1.00	*---*
	≥150	324 (61.0)	194 (60.1)	1.04 (0.78, 1.38)	0.782	1.04 (0.78, 1.39)	0.790
**Mobile-phone service** (years)	<12	242 (45.6)	144 (44.6)	1.00	*---*	1.00	*---*
	≥12	289 (54.4)	179 (55.4)	0.96 (0.73, 1.27)	0.778	1.00 (0.75, 1.34)	0.982

a Adjusted for age, gender, cigarette smoking, tea drinking, BMI, and work pressure.

b The cut points of tertiles of daily occupational exposure time (DOET) were ≤1.5 hours/day for T1, 1.5< DOET ≤4 hours/day for T2, and >4 hours/day for T3.

Table 3 presented the associations of various EMF exposures with sleep duration. There was an increased risk of short sleep in the group for whom occupational exposure duration was 23 years or more (OR = 1.49, 95% CI: 0.99, 2.23), compared with participants whose occupational EMF exposure duration was less than 23 years, but it did not reach the significance threshold (*p = *0.054).

**Table 3:** Associations^a^ of sleep duration^b^ with various electromagnetic exposures.

Variables		Medium sleep,(N = 335)	Short sleep,(N = 207)	OR (95%CI)	*P* Value	Long sleep,(N = 312)	OR (95%CI)	*P* Value
**Daily Occupational** **Exposure Time** (hours)^c^	T1	118 (35.2)	59 (28.5)	1.00	*---*	105 (33.7)	1.00	*---*
	T2	113 (33.7)	74 (35.7)	1.38 (0.89, 2.14)	0.568	101 (32.4)	1.02 (0.70, 1.50)	0.634
	T3	104 (31.0)	74 (35.7)	1.52 (0.97, 2.39)	0.174	106 (34.0)	1.23 (0.83, 1.81)	0.259
**Occupational** **Exposure Duration** (years)	<23	184 (54.9)	88 (42.5)	1.00	*---*	175 (56.1)	1.00	*---*
	≥23	151 (45.1)	119 (57.5)	1.49 (0.99, 2.23)	0.054	137 (43.9)	1.24 (0.86, 1.79)	0.254
**Electric fee**(yuan/month)	<150	130 (38.8)	69 (33.3)	1.00	*---*	137 (43.9)	1.00	*---*
	≥150	205 (61.2)	138 (66.7)	1.27 (0.88, 1.83)	0.208	175 (56.1)	0.83 (0.60, 1.14)	0.249
**Mobile-phone** **service** (years)	<12	145 (43.3)	89 (43.0)	1.00	*---*	152 (48.7)	1.00	*---*
	≥12	190 (56.7)	118 (57.0)	0.89 (0.62, 1.28)	0.541	160 (51.3)	0.86 (0.62, 1.19)	0.357

a Adjusted for age, gender, cigarette smoking, tea drinking, BMI, and work pressure.

b Medium sleep indicates that sleep duration is between 7 and 8 hours, short sleep is less than 7 hours, and long sleep is more than 8 hours.

c The cut points of tertiles of daily occupational exposure time (DOET) were ≤1.5 hours/day for T1, 1.5 <DOET ≤4 hours/day for T2, and >4 hours/day for T3.

### Stratified analysis concerning the effect of daily occupational exposure time on sleep quality and sleep duration

The effect of the daily electromagnetic exposure time on sleep quality was also evaluated, after stratification by occupational exposure duration (Table 4). Compared with subjects who had shorter daily occupational electromagnetic exposure time, participants with longer daily electromagnetic exposure time in the long occupational electromagnetic exposure group (exposure duration > = 23 years) had an increased risk of pool sleep quality (T2: OR = 2.12, 95%CI: 1.23–3.66; T3: OR = 1.83, 95%CI: 1.07–3.15). As portrayed in Table 5, the association of daily occupational exposure time with sleep duration, stratified by occupational exposure duration, demonstrated no significant statistical difference. Additionally, the results among shift workers were very similar to those in the whole sample (data not shown).

**Table 4:** The associations^a^ of daily occupational exposure time (DOET)^b^ with sleep quality stratified by occupational exposure duration^c^.

DOET (hours)	Short Occupational Exposure Duration (years) (N = 447)			Long Occupational Exposure Duration (years) (N = 407)		
	No. (Poor Sleeper[%])	OR (95%CI)	*P* Value	No. (Poor Sleeper[%])	OR (95%CI)	*P* Value
**T1**	102 (55.1)	1.00	*---*	48 (49.5)	1.00	*---*
**T2**	91 (65.0)	1.34 (0.82, 2.18)	0.243	103 (69.6)	2.12 (1.23, 3.66)	**0.007**
**T3**	77 (63.1)	1.30 (0.79, 2.15)	0.308	110 (67.9)	1.83 (1.07, 3.15)	**0.028**

a Adjusted for age, gender, cigarette smoking, tea drinking, BMI, and work pressure.

b The cut points of tertiles of daily occupational exposure time (DOET) were ≤1.5 hours/day for T1, 1.5 <DOET ≤4 hours/day for T2, and >4 hours/day for T3.

c Short occupational exposure duration means less than or equals 23 years, while long occupational exposure duration means more than 23 years.

**Table 5:** The associations^a^ of daily occupational exposure time (DOET)^b^ with sleep duration stratified by occupational exposure duration^c^.

	Short Occupational Exposure Duration (years) (N = 447)					
	Short sleep duration			Long sleep duration		
DOET (hours)	N (%)	OR (95%CI)	*P* Value	N (%)	OR (95%CI)	*P* Value
**T1**	38 (20.5)	1.00	*---*	64 (34.6)	1.00	*---*
**T2**	26 (18.6)	1.09 (0.58, 2.08)	0.922	57 (40.7)	1.24 (0.74, 2.07)	0.924
**T3**	24 (19.7)	1.28 (0.66, 2.48)	0.518	54 (44.3)	1.60 (0.93, 2.74)	0.139
	**Long** **Occupational Exposure Duration (years) (N = 407)**					
	**Short sleep duration**			**Long sleep duration**		
**DOET (hours)**	**N (%)**	**OR (95%CI)**	***P*** ** Value**	**N (%)**	**OR (95%CI)**	***P*** ** Value**
**T1**	21 (21.7)	1.00	*---*	41 (42.3)	1.00	*---*
**T2**	48 (32.4)	1.37 (0.70, 2.71)	0.605	44 (29.7)	0.66 (0.36, 1.23)	0.330
**T3**	50 (30.9)	1.44 (0.72, 2.85)	0.446	52 (32.1)	0.72 (0.39, 1.33)	0.639

a Adjusted for age, gender, cigarette smoking, tea drinking, BMI, and work pressure.

b The cut points of tertiles of daily occupational exposure time (DOET) were ≤1.5 hours/day for T1, 1.5 <DOET ≤4 hours/day for T2, and >4 hours/day for T3.

c Short occupational exposure duration means less than or equals 23 years, while long occupational exposure duration means more than 23 years.

## Discussion

The present study demonstrated a positive association of daily electromagnetic exposure time with the risk of poor sleep quality, but not with the risk of sleep duration. Moreover, individuals with longer daily electromagnetic field exposure time in the long-term occupational exposure group had an augmented hazard of sleep disturbances. On the contrary, there were no statistically significant associations of years of mobile-phone service, electric fee per month or occupational exposure duration with sleep quality and sleep duration.

The International Agency for Research on Cancer (IARC) classifies electromagnetic fields as ‘possible human carcinogens’ that might transform normal cells into cancer cells [27]. Owing to the high utilization of electricity in day-to-day life, exposure to power-frequency EMFs is unavoidable. Our study found that longer daily occupational exposure time may exert a hazard effect on sleep quality. There were seldom relative studies investigating the association of sleep quality and occupational exposure in power plants. But two surveys showed that the prevalence of difficulties with falling and remaining asleep increased with increasing short-wave frequency magnetic field exposure [28], [29]. Both panel studies denoted that sleep quality improved after interruption of the exposure. In addition, Loughran et al [22] reported that exposure to electromagnetic fields emitted by digital mobile-phone handsets prior to sleep decreased the rapid eye movement (REM) sleep latency and increased the electroencephalogram spectral power in the 11.5 to 12.25 Hz frequency range during the initial part of sleep following exposure. It indicated that mobile phone exposure prior to sleep may promote rapid eye movement sleep, which yielded to our study. Nevertheless, we did not observe significant associations of monthly electric fee or years of mobile phone service with sleep quality and sleep duration. This may owe to the accuracy of daily occupational electromagnetic exposure time to measure the intensity of electromagnetic exposure. An experiment on healthy subjects also found that there were no significant effects of electromagnetic field exposure on night sleep [30]. With their results from sleep EEG (Electroencephalography) data, they found that in the non-REM sleep, dimensional complexity decreased when sleep became deeper. During the REM sleep, they observed high dimensional values, indicating the increased information process.

Environmental RF-EMF sources like mobile phone base stations or W-LAN access points produce a continuous but lower and more homogenous exposure to the whole body. In the present study, in the group of long occupational electromagnetic radiation exposure duration, daily occupational electromagnetic exposure time enhanced the odds of poor sleep quality. Conversely, an Austrian survey, which focused on subjective symptoms, sleep problems and cognitive performance of people living near mobile phone base stations, found that sleep quality was not related to electromagnetic exposure [23]. A Swiss study also did not indicate an impairment of subjective sleep quality due to exposure from various sources of RF-EMFs in everyday life [31]. The 10% most exposed participants had an estimated risk for sleep disturbances of 1.11 (95%CI: 0.50 to 2.24).

The studies regarding the probable mechanism of the effect of electromagnetic radiation on sleep focused on the regulation of melatonin. Melatonin is a natural hormone produced by pineal gland activity in the brain that regulates the body’s sleep wake cycle. Although a change in melatonin synthesis with exposure to magnetic fields has been reported for a variety of experimental animal models [32], [33], only a few studies have attempted to determine whether such effects occur in humans. Altpeter et al. [29] found evidence that EMF exposure was associated with sleep quality and melatonin excretion, which was only in poor sleepers. Moreover, Wilson et al [34] found a reduction in nocturnal urinary concentrations of the major melatonin metabolite 6-hydroxymelatonin sulfate (6-OHMS) in some persons after 8 weeks of extremely low frequency electric or magnetic exposure. A reduction of 6-OHMS excretion in early evening, but not overnight, was reportedly in a study of railway workers with occupational exposure to 16.7 Hz magnetic fields [35]. Among the electric utility workers, magnetic field intensity, intermittence, or cumulative exposure had little influence on nocturnal 6-OHMS excretion [36]. On the other hand, another possible mechanism may involve the modulation of regional cerebral blood flow (rCBF) after exposure to mobile phones [37]. There was an increase in relative rCBF in the dorsolateral prefrontal cortex on the side of exposure.

The strength of the present study is that we assess the electromagnetic radiation exposure in the workers of a power plant rather than the general population in which is hard to distinguish the degree of exposure. Our study also has several limitations. Firstly, this study is a cross-sectional study. Recall bias would be introduced into assessments of exposures and outcomes. Secondly, we cannot rule out the high percentage of missing data. But the distribution of socio-demographic characteristics between subjects with and without missing data was comparable. Thirdly, the subjective sleep parameters, including sleep quality and sleep durations, might be considered a weakness of this study. But several reports have found that self-reported data on sleep status are comparable with physiologic data to a certain degree [38], and a study has shown that this single question was a valid indicator of integrated sleep status [39]. Although shift work was correlated with circadian disruption [40], the associations of EMF exposure with poor sleep quality had no change when we did the analysis in the shift workers. In addition, there was uncertainty and potential bias in electromagnetic field exposure assessment, due to the potential tendency for the cases to over-report their electromagnetic usage relative to controls. Although the RF field can be measured, individual exposure from environmental electromagnetic radiation produced in the home or workplace is difficult to assess.

## Conclusions

In conclusion, our study suggests that poor sleep quality is significantly associated with the daily electromagnetic field exposure time in an electric power plant. It implies EMF exposure may damage human sleep quality rather than sleep duration. However, these results should be further confirmed in the future.
